# Historical and Contemporary Factors Govern Global Biodiversity Patterns

**DOI:** 10.1371/journal.pbio.1001294

**Published:** 2012-03-27

**Authors:** Jonathan Chase

**Affiliations:** Freelance Science Writer, Saint Louis, Missouri, United States of America

## Abstract

A novel hierarchical framework integrates the effects of time, area, productivity, and temperature at their respective relevant scales and successfully predicts the latitudinal gradient in global vertebrate diversity.

**Figure:**
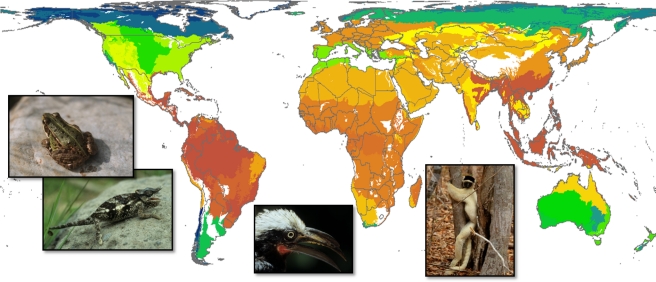
Global patterns of terrestrial vertebrate diversity analyzed in the study. Each of the 32 bioregions is colored by its vertebrate species richness (amphibian, reptile, bird, mammal richness combined; dark green represents the lowest values and dark red represents the highest values).

In one of the greatest episodes of a “scoop” in scientific history, Alfred Russell Wallace's co-discovery of evolution by natural selection has relegated him to a perpetual footnote to Charles Darwin's transformative theory. However, Wallace and Darwin, along with a handful of other 19th century naturalists, provided more than just insights about how species changed through evolutionary time. In their respective voyages replete with tropical disease, run-ins with natives, and a variety of ship-related mishaps, these men gathered meticulous data on all creatures great and small, and then spent decades synthesizing their observations to understand not only how life evolved, but how it was variously distributed across the planet.

Despite more than 150 years of intense investigation since, “what determines species diversity” remains one of the top 25 research challenges in all of science according to a 2005 feature in *Science* magazine. One reason for the lack of clarity is the great chasm in scales by which biodiversity is studied; large-spatial and long-temporal scales are studied in the realm of evolutionary biology and biogeography, and smaller spatio-temporal scales are studied in the realm of ecology.

Standing on the shoulders of the countless masses of scientists whose careful cataloging and observation has led to unprecedented information on the distribution and abundance of species, the contemporary biodiversity scientist no longer needs passage on voyaging ships and a whole lot of chutzpah, but rather needs to be adept at analyzing and interpreting tens of thousands of data points using sophisticated computational and analytical tools. In this month's issue of *PLoS Biology*, Jetz and Fine take an analytical leap forward in biodiversity research by simultaneously comparing the relative importance of long-term, large-scale processes and contemporary and smaller-scale processes in driving patterns of the biodiversity among the four groups of terrestrial vertebrates (amphibians, reptiles, birds, and mammals).

Although a multitude of processes, including history, habitat area, and productivity, have been implicated as important drivers of biodiversity at multiple spatial scales, Jetz and Fine's analysis is the first to put them together into a single statistical framework. By doing so, they can explore just how important the various processes underlying biodiversity are, and at which scale their importance is manifest.

First, Jetz and Fine divided the globe into 32 well-defined terrestrial bioregions, including each of the major biomes (e.g., deserts, grasslands, tropical forests) separated according to their geographic location. A habitat's areal extent can strongly influence its biodiversity; habitats with more area have a higher likelihood of diversification and are more heterogeneous, providing more available niches. But when the authors used the modern-day area covered by each of their bioregions as a predictor of the number of species, they found a rather poor fit.

Next, the authors incorporated the geological history of these bioregions into the biodiversity equation. To do so, they calculated the change in areal extent of each bioregion over the last 55 million years, as the climate cooled and dried. For example, though grasslands cover a large proportion of the earth today, this extent is relatively new (less than 8 million years old) and there has not been enough time for species to diversify in proportion to the current available area. Tropical forests, however, previously covered a much greater area and have had much more time for diversification. With the hindsight of history, the model's predictive ability improved considerably.

In addition to habitat area, these bioregions differ in several other important features, most notably productivity (the rate of carbon fixed by photosynthesis by the flora as a result of precipitation and radiant energy) and temperature. Tropical forests are more productive than deserts, for example, and this can greatly influence biodiversity by increasing the numbers of individuals and thus likelihood of diversification, as well as by increasing the numbers of niches for those species to inhabit. By including productivity and temperature into the model, the authors found another leap in predictability; they were able to explain more than 80% of the variability in biodiversity among these bioregions with just these few predictor variables.

Having established the primacy of historical area, productivity, and temperature in determining biodiversity at the bioregion scale, the authors took off their biogeographer hats and put on their ecologist hats to evaluate how biodiversity is partitioned into local communities (110-km grid cells). Although a decent proportion (30%–60%) of the variance in smaller scale biodiversity could be explained simply by knowing how many species occurred in the bioregion, incorporating the local productivity of the grid cell improved the model's predictability considerably, suggesting a role for ecological interactions in determining which, and how many, species live in a given locality.

By integrating habitat area, history, and productivity at multiple spatial scales into a single hierarchical framework, Jetz and Fine gain a clearer understanding of the drivers of biodiversity than has been accomplished previously. There is no doubt that we are in the midst of the sixth mass extinction event on this planet and the cause is us. By achieving greater understanding of the underlying causes and correlates of current-day biodiversity, this analysis can also help point the way towards a deeper understanding of how our activities, by destroying habitats and changing climate, may continue to alter patterns of biodiversity in the future. And perhaps, point us towards ways that we might be able to stave off some of these changes.

**Jetz W, Fine PVA (2012) Global Gradients in Vertebrate Diversity Predicted by Historical Area-Productivity Dynamics and Contemporary Environment. doi:10.1371/journal.pbio.1001292**

